# Construction of cell factory through combinatorial metabolic engineering for efficient production of itaconic acid

**DOI:** 10.1186/s12934-022-02001-1

**Published:** 2022-12-28

**Authors:** Jiao Feng, Chunqiu Li, Hao He, Sheng Xu, Xin Wang, Kequan Chen

**Affiliations:** 1 State Key Laboratory of Materials-Oriented Chemical Engineering, College of Biotechnology and Pharmaceutical Engineering, Nanjing Tech University, No. 30 Puzhu Road(S), Nanjing, 211816 People’s Republic of China; 2 Petrochemical Research Insitute of Petrochina Co. Ltd., Beijing, 102206 People’s Republic of China

**Keywords:** Itaconic acid, *E. coli* catalysis, Enzyme evolution, Protein scaffolds, Pathway optimization

## Abstract

**Background:**

Itaconic acid, an unsaturated C5 dicarbonic acid, has significant market demand and prospects. It has numerous biological functions, such as anti-cancer, anti-inflammatory, and anti-oxidative in medicine, and is an essential renewable platform chemical in industry. However, the development of industrial itaconic acid production by *Aspergillus terreus,* the current standard production strain, is hampered by the unavoidable drawbacks of that species. Developing a highly efficient cell factory is essential for the sustainable and green production of itaconic acid.

**Results:**

This study employed combinatorial engineering strategies to construct *Escherichia coli* cells to produce itaconic acid efficiently. Two essential genes (cis-aconitate decarboxylase (CAD) encoding gene *cadA* and aconitase (ACO) encoding gene *acn*) employed various genetic constructs and plasmid combinations to create 12 recombination *E. coli* strains to be screened. Among them, *E. coli* BL-CAC exhibited the highest titer with citrate as substrate, and the induction and reaction conditions were further systematically optimized. Subsequently, employing enzyme evolution to optimize rate-limiting enzyme CAD and synthesizing protein scaffolds to co-localize ACO and CAD were used to improve itaconic acid biosynthesis efficiency. Under the optimized reaction conditions combined with the feeding control strategy, itaconic acid titer reached 398.07 mM (51.79 g/L) of engineered *E. coli* BL-CAR470E-DS/A-CS cells as a catalyst with the highest specific production of 9.42 g/g_(DCW)_ among heterologous hosts at 48 h.

**Conclusions:**

The excellent catalytic performance per unit biomass shows the potential for high-efficiency production of itaconic acid and effective reduction of catalytic cell consumption. This study indicates that it is necessary to continuously explore engineering strategies to develop high-performance cell factories to break through the existing bottleneck and achieve the economical commercial production of itaconic acid.

**Supplementary Information:**

The online version contains supplementary material available at 10.1186/s12934-022-02001-1.

## Background

Itaconic acid, an unsaturated C5 dicarbonic acid, is an essential renewable platform chemical in the industry [1–3]. As a potential substitute for some petrochemical-based monomers, it has been widely used to produce of polymers and materials, such as polyesters, synthetic latex, fiber, resins, plastic, and methyl methacrylate [3–5]. Its biological functions have recently drawn increasing attention in anti-microbial, anti-viral, anti-cancer, anti-inflammatory, anti-oxidative, and nutrition regulation [6–8]. With the increasing total market demand annually, there is a great need to develop an efficient method to produce itaconic acid.

The chemical process was the first reported method of synthesizing itaconic acid [9]. Still, itaconic acid’s low yields and high costs limited its commercial practice and large-scale expansion [5, 9, 10]. Biosynthesis is a suitable choice and has been applied to the industrial production of itaconic acid by fermentation of *Aspergillus terreus.* After years of systematic optimization, itaconic acid produced by *A. terreus* fermentation has increased from the initially reported 27 g/L to 160 g/L with a productivity of 1.0 g/(L·h) [7, 11, 12]. However, it is hindered by the significant gap between the actual level and the expected theoretical maximum titer of 240 g/L due to the limitations of physiological properties of *A. terreus*, such as poor growth, high sugar consumption, sensitivity to the oxygen supply, long duration of fermentation, uncontrollable byproducts [5, 13, 14]. Thus, many efforts focus on developing other organisms and strategies for producing itaconic acid. Some native strains, like *Ustilago maydis*, *Pseudozyma*, *Candida*, et al. [15–17], and engineered strains, like *Saccharomyces cerevisiae*, *Escherichia coli*, *Corynebacterium glutamicum*, et al. [13, 18, 19], have been developed to produce itaconic acid. However, their production capacities of itaconic acid by fermentation are still lower than *A. terreus* [5]*.*

Compared with fermentation, a whole-cell catalytic system was developed to convert citric acid to itaconic acid and showed advantages in producing itaconic acid, including low cost, short process time, easy preparation, and rapid conversion [5, 20]. Recently, Kim et al. first developed a whole-cell system based on the engineered *E. coli* expressing aconitase (ACO) and cis-aconitate decarboxylase (CAD), which produced itaconic acid of 319.8 mM (41.6 g/L) from 500 mM citrate with 64.0% conversion [20]. Developing a high-performance cell factory as a biocatalyst is the key solution to solving the problem of the low production efficiency of itaconic acid. Some scientists have engineered other microorganisms as the hosts, including *Shewanella livingstonensis* Ac10 and *Halomonas bluephagenesis* TD01, and taken other facilitation measures, such as heat treatment and metabolic engineering [5, 21]. It is worth noting that the systematic engineering of strains by metabolic engineering strategies can efficiently improve biocatalyst performance [22]. Some strategies and techniques have been successfully used to construct industrially competitive strains, such as gene expression modulation, enzyme evolution, and protein assembly [22, 23]. However, the studies on meticulous and systematic strategies of the engineering strain for itaconic acid production are few in  comparison to those on other chemicals. It is necessary to explore more optimization strategies, such as protein engineering and substrate channeling, to improve cellular catalytic efficiency for itaconic acid production.

Here, the biosynthesis pathway of itaconic acid was constructed in *E. coli* cells and was enhanced using a variety of genetic constructs and plasmid combinations. Adopting varied copy number plasmids to modulate gene expression levels, employing enzyme evolution to optimize rate-limiting enzyme CAD, and synthesizing protein scaffolds to co-localize pathway enzymes (ACO and CAD) were used to develop high-performance recombinant *E. coli* cells to improve itaconic acid biosynthesis. In addition, reaction conditions were optimized and combined with regulatory strategies that resulted in a high titer of itaconic acid with a smaller amount of recombinant *E. coli.*

## Results and discussion

### Construction of E. coli cells for itaconic acid production via different genetic strategies

To construct the *E. coli* cells as biocatalysts for itaconic acid production, *acn* from *C. glutamicum* and *cadA* of *A. terreus* were co-expressed in *E. coli* strain BL21 (DE3). For heterologous gene expression, various factors, such as plasmid copy number, promoter strength, and gene construct (such as the number, source, or order of genes), can lead to differential expression of multiple genes and affect the production of recombinant proteins and products [23–25]. Koffas et al. have reconstructed the synthesis pathway of catechins in *E. coli* cells containing different gene constructs through screening the source of genes and modulation of gene number on a single plasmid resulting in a significant difference in the biosynthesis capability of catechins [23]. The control of gene expression is critical for the efficiency of multistep metabolic engineering. This study used a series of double cistronic vectors with varying copy numbers from 10 to 100 to reconstruct the itaconic acid synthesis pathway in *E. coli* cells to adjust *acn* and *cadA* expression levels.

**Fig. 1:**
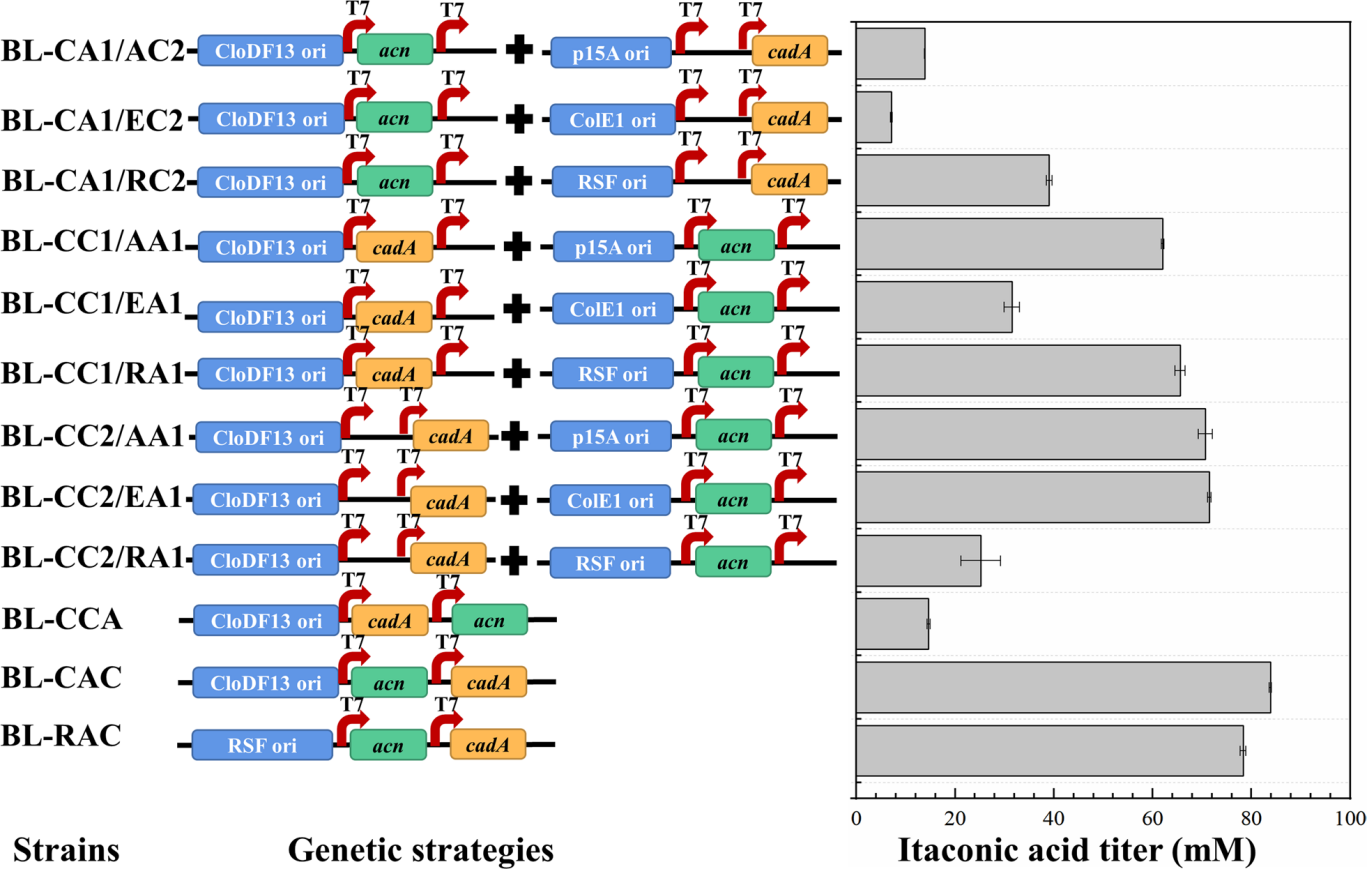
Schematic diagram and itaconic acid titer of recombinant strains with different genetic strategies

To obtain stable and efficient whole-cell biocatalysts, twelve recombinant *E. coli* strains were constructed according to the genetic strategies based on various genetic constructs and plasmid combinations (Fig. 1). By evaluating the effects of *acn* and *cadA* co-expression in one or two plasmids, the highest itaconic acid titer of 83.84 mM was produced by *E. coli* BL-CAC containing pCDF-acn-cadA at 12 h when 100 mM of citrate was used as substrate (Fig. 1). However, the strain *E. coli* BL-CCA containing pCDF-cadA-acn showed deficient itaconic acid production. Distinct yield gaps in itaconic acid caused by gene insertion into different multiple cloning sites (MCS) suggested differential expression of *acn* and *cadA* under different genetic strategies. Meanwhile, the BL-CC2/AA1 and BL-CC2/EA1 as whole-cell biocatalysts produced higher itaconic acid than the BL-CC1/AA1 and BL-CC1/EA1, respectively. These results indicated that the construction form of *acn* in MCS1 and *cadA* in MCS2 in a plasmid was suitable for itaconic acid production.

The apparent differences in itaconic acid production between the strains BL-CA1/AC2, BL-CA1/EC2, BL-CA1/RC2, BL-CC2/AA1, BL-CC2/EA1, and BL-CC2/RA1 indicated that gene copy number significantly affected the efficiency of the constructed itaconic acid synthesis pathway. Kim et al. reported that boosting the copy number of *cadA* could improve citrate conversion, while other recent studies showed that the expression of ACO played a crucial role in itaconic acid synthesis [5, 20]. Our study constructed pRSF-acn-cadA further to increase the copy number of *cadA* and *acn.* However, BL-RAC did not exhibit a further improvement in itaconic acid titer over BL-CAC as expected. It might be due to no further increase in the expression of ACO and CAD. In conclusion, these data indicated that *E. coli* BL-CAC containing pCDF-acn-cadA was superior for itaconic acid production and was selected for further study.

### Optimization of itaconic acid production by whole-cell biocatalysis

**Fig. 2:**
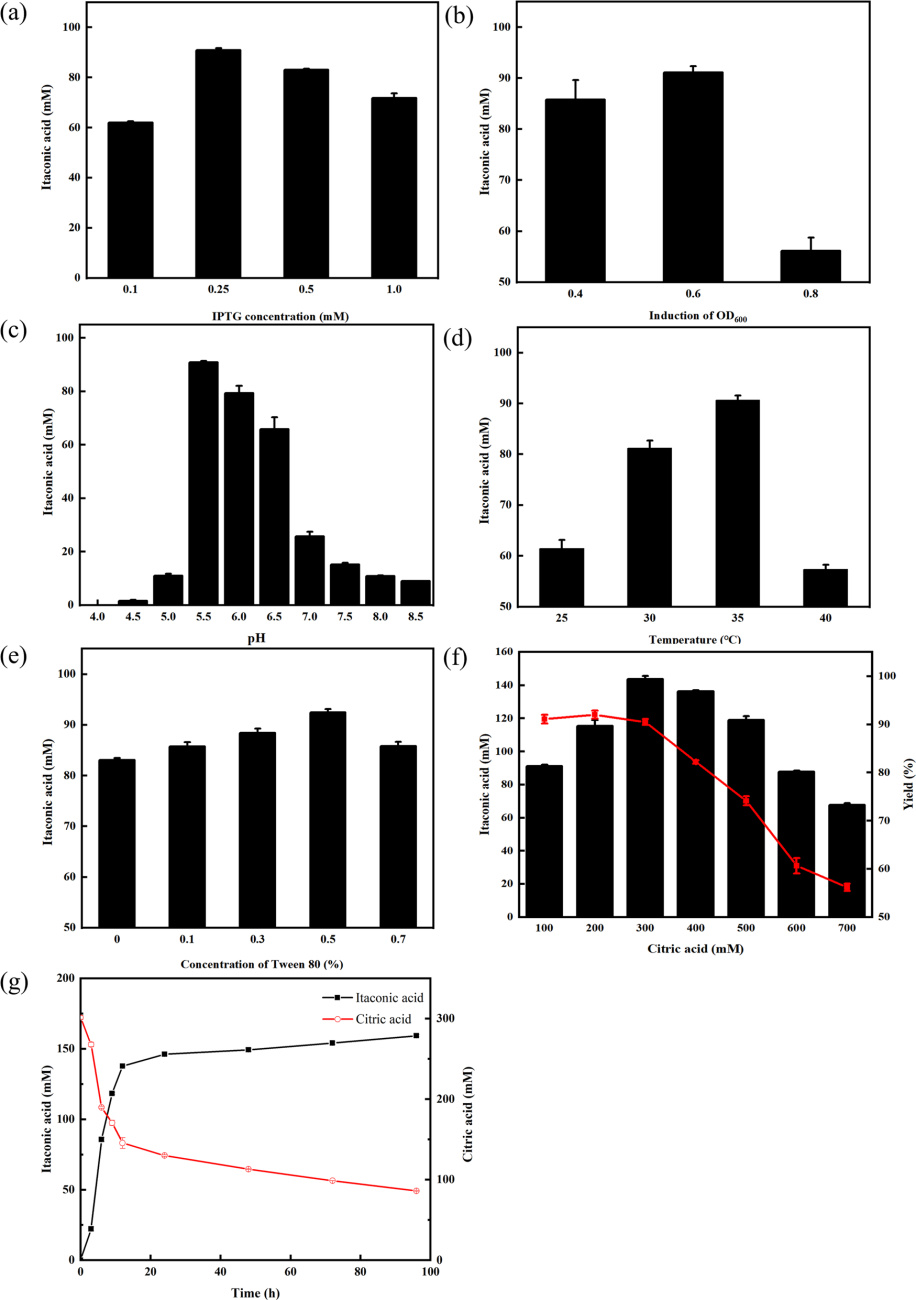
Optimization of the synthesis conditions of itaconic acid by the recombinant *E. coli* BL-CAC. The effects of IPTG **a**; induction of OD_600_ **b**; reaction pH (**c**); reaction temperature **d**; concentration of Tween 80 **e**; concentration of substrate **f**; and time profiles of itaconic acid production **g**

Induction and reaction conditions are the critical factors for the activity of biocatalysts and catalytic efficiency, affecting itaconic acid production. Under the control of the T7 promoter, *acn* and *cadA* expressions were generally induced by IPTG. The induction efficiencies generally related to the soluble expression of heterologous proteins might be regulated by IPTG concentration, incubation temperature, and incubation OD_600._ Thus, the effects of different induction temperatures (18 °C, 25 °C, and 30 °C), IPTG concentration from 0.1 to 1 mM, and different incubation OD_600_ (0.4, 0.6, and 0.8) were investigated. As observed on SDS-PAGE (Additional file 1: Figure S1), CAD and ACO were primarily expressed as insoluble inclusion bodies at 25 °C and 30 °C, which was consistent with previous reports [20]. However, the formation of inclusion bodies was significantly reduced at induction temperatures of 18 °C, so the lower temperature was beneficial for the soluble production of ACO and CAD (Additional file 1: Figure S1). By comparing the titers of itaconic acid, the optimal IPTG concentration was 0.25 mM, and the induction time was an OD_600_ of 0.6 (Fig. 2a, b). A lower expression level at low temperature and inducer concentration might result in a slower translation rate, which favors proper folding to decrease the formation of inclusion bodies [26, 27].

Furthermore, reaction pH, temperature, Tween 80 concentration, and substrate concentration were investigated to optimize the catalytic efficiency of *E. coli* BL-CAC. Various pH (4.0 to 8.5) and reaction temperatures (25–40 °C) were used for the assessment of itaconic acid production, which peaked at pH 5.5 and 35 °C, respectively (Fig. 2c, d). To improve the transport of substrate and product, the surfactant Tween 80 was supplemented to increase cell membrane permeability. As shown in Fig. 2e, relative itaconic acid titer increased with the increasing Tween 80 from 0 to 0.5% and decreased after 0.5%. Finally, substrate concentration was optimized to obtain a high level of itaconic acid production (Fig. 2f). High yields could be achieved when the citrate concentration was in the range of 100 to 300 mM. Among them, the itaconic acid titer was the highest at the citrate concentration of 300 mM, indicating that 300 mM citrate was acceptable for itaconic acid production.

Under the optimal induction condition, the recombinant *E. coli* BL-CAC was cultivated and used as the biocatalyst to exhibit the highest catalytic efficiency in the optimized reaction system. As the results illustrated in Fig. 2g, itaconic acid titer was accumulated to 159.27 mM at 96 h. Production of itaconic acid and the consumption of citrate increased rapidly in the first 12 h, with the highest yield (mol/mol) of 90.47% at 9 h, and then tended to be slow, with the molar yield dropping below 80% after 48 h. In the meantime, accumulations of the intermediate cis-aconitate and its isomer, trans-aconitate, were detected. During the two-step reaction, trans-aconitate isomerized from cis-aconitate was not catalyzed by CAD, resulting in low conversion efficiency. Therefore, further efforts are still needed to improve production.

### Engineering the rate-limiting enzyme CAD

**Fig. 3:**
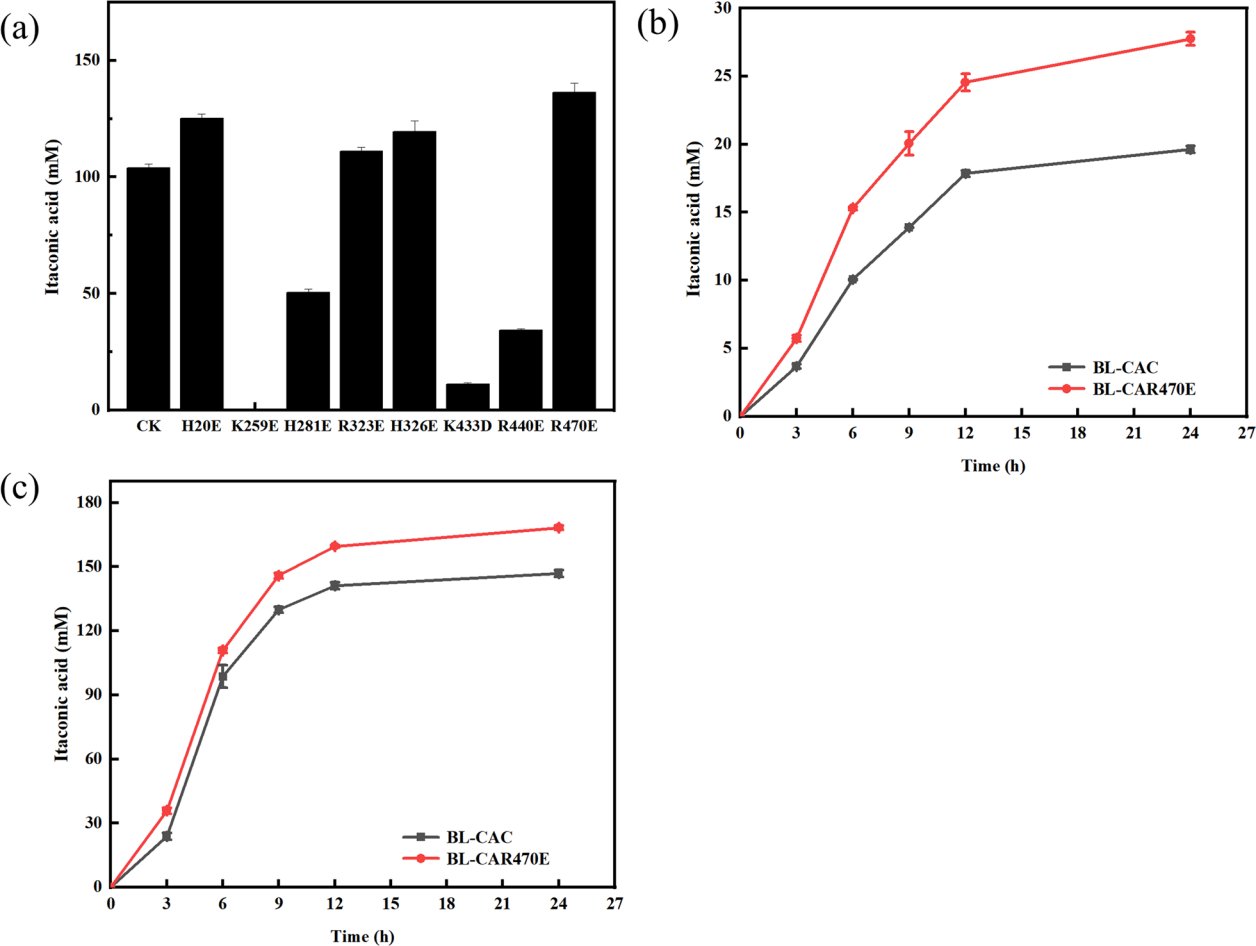
The itaconic acid production of CAD mutants obtained by site-directed mutagenesis **a**. *E. coli* BL-CC is used as a control check (CK); Comparison of itaconic acid titer between recombinant *E. coli* BL-CAC and BL-CAR470E at pH 5.0 **b** and pH 5.5 **c**

Accumulations of the intermediates suggested that CAD was a rate-limiting enzyme for itaconic acid production [20, 28]. Meanwhile, we found that the CAD activity decreased markedly (almost 0) at pH 5.0, consistent with previous reports [29]. The input of the substrate citric acid led to the decrease of reaction pH, resulting in the inhibition or even inactivation of CAD. A large amount of alkali needed to add initially, increasing the cost and post-processing. To improve the acid resistance and catalytic activity of CAD, eight mutation sites were screened and designed as H20E, K259E, H281E, R323E, H326E, K433D, R440E, and R470E through the multiple sequence alignments and Weblogo analysis of CAD (Additional file 1: Figure S2, Table S3). As shown in Fig. 3a, four dominant single-point mutants, H20E, R323E, H326E, and R470E, improved the catalytic efficiency of CAD in the whole-cell reaction with cis-aconitate as the substrate at pH 5.0, and the optimal mutant was determined to be R470E. Further co-catalyzed with the former enzyme ACO, the mutant strain BL-CAR470E was constructed, and itaconic acid titers increased by 41.3% and 14.7% at pH 5.0 and pH 5.5 at 24 h, respectively (Fig. 3b, c). The results suggested that mutant R470E effectively improved the acid resistance and catalytic activity of CAD. However, there was a big gap between the catalytic titer of itaconic acid under the acidic condition (pH 5.0) and that under the optimal condition (pH 5.5), which indicated that further optimization was needed. Through molecular simulation, it was speculated that amino acid R470 and catalytic key amino acid H111 are in the same channel. The mutation of R470 to E470 caused the nearby spatial structure of the H111 site to shift and change, and the substrate channel’s shape was directly affected, thereby positively affecting the catalytic activity of CAD.

### Construction of scaffold-based protein complex in vivo to improve itaconic acid production

**Fig. 4:**
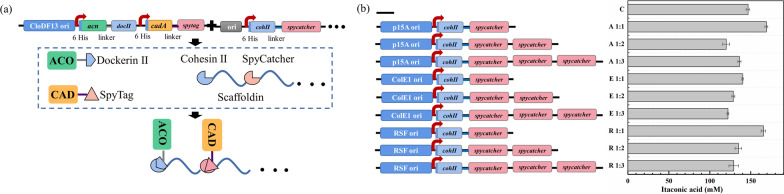
Schematic illustration on enzyme assembly **a**; The effect of various scaffoldins on itaconic acid production **b**. The free-scaffold strain *E. coli* BL-CAC was used as a control strain **C**

Substrate channeling engineered by direct fusion, synthetic protein scaffold, or bacterial microcompartment is an effective strategy to enhance metabolic fluxes [22]. Synthetic protein scaffold can colocalize multiple enzymes into a multi-enzyme complex via orthogonal interaction domains [30]. The scaffold-based protein complex brings enzymes in spatial proximity to facilitate substrate channeling between active sites, which shortens intermediates’ transit times and prevents intermediates’ loss and instability [31]. In order to relieve the loss of cis-aconitate and improve the synthesis efficiency of itaconic acid, the two-enzyme (ACO and CAD) complex was assembled in vivo using the synthetic protein scaffold based on two interaction pairs Cohesin II-Dockerin II and SpyCatcher-SpyTag. The scaffoldin was composed of Cohesin II and SpyCatcher, which controlled the stoichiometry of two enzymes. ACO and CAD were fused with Dockerin II and SpyTag, which recruited ACO and CAD to the scaffoldin by specific interaction between the Cohesin II and Dockerin II, SpyCatcher and SpyTag (Fig. 4a). We first tested whether two interaction pairs Cohesin II-Dockerin II and SpyCatcher-SpyTag can achieve enzyme assembly. The indistinguishable itaconic acid titer of BL-CA-DII-C-ST and BL-CAC suggested that ACO-Dockerin II and CAD-SpyTag retained all catalytic activity compared to the unfused proteins (Additional file 1: Figure S3). SDS-PAGE analysis showed that ACO-Dockerin II, CAD-SpyTag, and the Cohesin II-SpyCatcher scaffoldin were expressed and purified normally. The native PAGE analysis exhibited a new high-molecular-weight band in the mixture (Additional file 1: Figure S4). These results indicated that the expression and structure of ACO-Dockerin II and CAD-SpyTag were not compromised, and then the scaffoldin could bind ACO and CAD to form the two-enzyme complex.

Subsequently, the fusion enzymes expression plasmid and scaffoldin plasmid were simultaneously transformed into *E. coli* BL21 (DE3) to investigate the effect of synthetic protein scaffold complex on itaconic acid production. It is a property of synthetic protein scaffolds to control enzyme stoichiometry and thus regulate the enzyme levels for the target products [31]. To optimize the production of itaconic acid, the number of SpyCatcher in scaffoldin was changed to regulate CAD level, and the expression of fusion pathway enzymes and scaffoldin was coordinated by varying copy numbers of the scaffoldin-encoding gene (Fig. 4b). Itaconic acid titer was higher in strains BL-CAC-DS/A-CS (15.37%) and BL-CAC-DS/R-CS (12.89%) containing the scaffold domains ratio of 1:1 compared with the scaffold-free strain, indicating that the two-enzyme complex formed by protein scaffold binding ACO and CAD could improve the production of itaconic acid. However, a further additional ratio of the CAD-binding domain did not increase itaconic acid production as expected. Moreover, the expression level of the scaffoldin resulted in a significantly different titer, which indicated an optimal combination in the protein scaffold complex for maximizing the pathway efficiency of itaconic acid production, including the ratio between the two pathway enzymes and their ratio to the scaffoldin [32, 33].

### Fed-batch strategy for itaconic acid production with recombinant E. coli BL-CAR470E-DS/A-CS cells

**Fig. 5:**
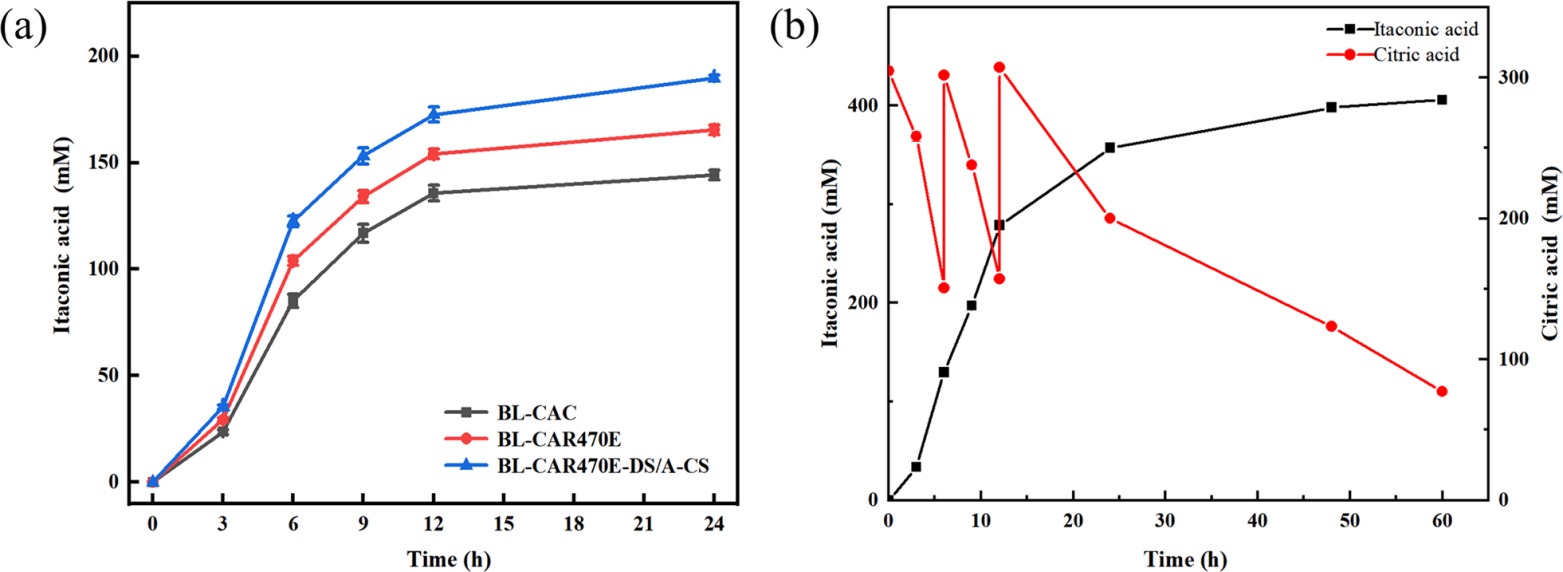
Comparison of itaconic acid titer between *E. coli* BL-CAC, BL-CAR470E, and BL-CAR470E-DS/A-CS **a**; Fed-batch strategy for the production of itaconic acid by *E. coli* BL-CAR470E-DS/A-CS **b**

Based on the above results, the recombinant strain BL-CAR470E-DS/A-CS was constructed to investigate the effect of mutant CAD combined with protein assembly on itaconic acid production. *E. coli* BL-CAR470E-DS/A-CS synthesized 172.56 mM (22.45 g/L) of itaconic acid at 12 h, and the maximum productivity reached 2.66 g/L/h, which was 43.94% higher than that of the starting strain BL-CAC (Fig. 5a). The intermittent feeding and pH control strategy further improved the itaconic acid titer to 398.07 mM (51.79 g/L) with a productivity of 1.08 g/L/h and a yield of 82.68% using 20 OD_600_ cells as the catalyst at 48 h (Fig. 5b). The specific production of itaconic acid was 9.42 g/g_(DCW)_.

Compared with the previous whole-cell catalytic production of itaconic acid, the results on the titer, productivity, specific production, biomass, and reaction time are summarized in Table 1. Using a large biocatalyst relative to the low substrate often leads to good catalytic results, such as high productivity or yield. For example, Kim et al. used 87 g/L cells in a whole-cell reaction to obtain 41.6 g/L with the highest productivity of 2.19 g/L/h [20], while Luo et al. used 50 mM citric acid in a whole-cell reaction with 35 g/L catalytic cells to get the highest yield of 110.69% [21]. We also found that using the low substrate concentration of 100 mM could achieve a high yield and conversion (greater than 90%). Increasing substrate concentration is necessary for industrial production but often inhibits the biocatalytic activity of cells [5]. A large number of catalysts undoubtedly increased the investments. Therefore, it is required to develop high-performance cell factories with high catalytic capacity per unit biomass. We employed combinatorial engineering strategies to construct *E. coli* BL-CAR470E-DS/A-CS cells for the efficient production of itaconic acid. The titer and the specific production of itaconic acid in this study were significantly increased compared with the previously engineered *E. coli* JY001 by Kim et al. [20]. Among whole-cell catalysis in heterologous cells, the specific production of itaconic acid in our study was the highest. The itaconic acid titer was higher than most others reported but lower than that reported by Zhang et al. [5], which might be the lower amount of biocatalyst input and unoptimized feeding control process. The highest specific production of itaconic acid among heterologous hosts indicated that the engineered *E. coli* BL-CAR470E-DS/A-CS had an excellent catalytic performance for itaconic acid production.

**Table 1:** Itaconic acid production of the engineered strains by whole-cell biocatalysis

Strain	Itaconic acid titer (g/L)	Productivity (g/L/h)	Specific production of itaconic acid (g/g _(DCW)_)	Biomass in reaction	Time (h)	Reference
*E. coli* JY001	41.60	2.19	0.48	87 g/L	19	20
*S. livingstonensis Ac10*	6.82	0.27	0.19	35 g/L	25	21
*H. bluephagenesis* TDZI-08	63.60	1.12	6.77	9.39 g/L^a^ (30 OD_600_)	54	5
*E. coli* BL-CAR470E-DS/A-CS	51.79	1.08	9.42	5.50 g/L^a^ (20 OD_600_)	48	This study

^a^The dry cell weights (DCW) of *H. bluephagenesis* and *E. coli* were determined by converting the OD_600_ with the coefficients of 0.313 g _(DCW)_ per (L·OD_600_) and 0.275 g_(DCW)_ per (L·OD_600_), respectively [35, 36]

## Conclusions

For highly efficient biosynthesis of itaconic acid, the recombinant itaconic acid biosynthesis pathway was constructed in *E. coli* according to different genetic strategies, with *E. coli* BL-CAC showing the highest activity. Then employing enzyme evolution to optimize rate-limiting enzyme CAD and synthesizing protein scaffolds to co-localize ACO and CAD were used to improve itaconic acid biosynthesis efficiency. Under the optimized reaction conditions combined with a feeding control strategy, itaconic acid titer reached 398.07 mM (51.79 g/L) using 20 OD_600_ cells of engineered *E. coli* BL-CAR470E-DS/A-CS as a catalyst with the highest specific production among heterologous hosts at 48 h.

## Methods

### Bacterial strains and plasmid construction

All strains and plasmids used in this study are listed in Additional file 1: Table S1. Target genes *acn* and *cadA* were amplified by Polymerase Chain Reaction (PCR) using the respective primers listed in Additional file 1: Table S2 for plasmid construction. The genome of *C. glutamicum* and synthetic codon-optimized *cadA* of *A. terreus* were used as the respective template of *acn* and *cadA. *After the extraction and digestion with restriction sites, amplified products were cloned into pACYCDuet-1, pCDFDuet-1, pETDuet-1, and pRSFDuet-1 according to the genetic strategies (Fig. 1), respectively. Using the primers in Additional file 1: Table S2, a megaprimer PCR was conducted for the mutations [34]. The single-site mutagenic megaprimers were amplified in the first stage of PCR. Then the whole plasmids containing the mutation site were amplified using pCDFduet-cadA2 as the template and megaprimers in the second stage of PCR, purified after *Dpn*I treatment, and transformed into *E. coli* Trans1-T1. In order to construct the synthetic scaffold, genes encoding Cohesin II, Dockerin II, SpyTag, and SpyCatcher were synthesized, and plasmids, such as pCDFduet-acn-cadA and pRSFduet-1, were linearized by PCR using the respective primers listed in Additional file 1: Table S2. The G4S linker (GGGGSGGGGS) and HXF linker (GSGGSGVD) introduced by designing primers were used for connecting the enzymes to their corresponding Dockerin II and SpyTag. The 6*His was designed to insert at the N-terminus of the ACO, CAD, and Cohesin II-SpyCatcher for protein purification. The linearized plasmids and corresponding target fragments were mixed according to the ClonExpress II One Step Cloning Kit (Vazyme, Nanjing, CA) to obtain the recombination plasmids pCDFduet-acn-Dockerin II-cadA-SpyTag, pRSFduet-Cohesin II-SpyCatcher. Then recombinant plasmids pRSFduet-Cohesin II-SpyCatcher × 2 and pRSFduet-Cohesin II-SpyCatcher × 3 were obtained by further increasing the amount of Spycather using the pRSFduet-Cohesin II-SpyCatcher as a template as above. The gene fragments of Cohesin II-SpyCatcher, Cohesin II-SpyCatcher × 2, and Cohesin II-SpyCatcher × 3 were digested with *Nco* I and *Hind* III from pRSFduet-Cohesin II-SpyCatcher, pRSFduet-Cohesin II-SpyCatcher × 2, pRSFduet-Cohesin II-SpyCatcher × 3 and ligated to pACYCduet-1, or pETduet-1. The plasmid of pCDFduet-acn-Dockerin II-R470E-SpyTag was obtained by replacing the CAD fragment with R470E fragment to investigate the effect of mutant CAD combined with protein assembly on the itaconic acid production. The recombination plasmids were transformed into *E. coli* BL21 (DE3) to obtain the respective recombinant *E. coli* strains (Additional file 1:Table S1).

### Culture conditions

After overnight activation, all strains were inoculated into Luria–Bertani (LB) medium, and appropriate antibiotics, including spectinomycin (50 μg/mL), ampicillin (50 μg/mL), kanamycin (50 μg/mL), or chloramphenicol (34 μg/mL) were added whenever necessary. To improve the activity of biocatalysts, the induction conditions were optimized. *E. coli* strains were cultured to the optical density (OD_600_) of 0.4–0.8 at 37 °C with shaking at 200 rpm and induced under 18 °C, 25 °C, or 30 °C with the addition of 0.1–1.0 mmol/L isopropyl-beta-D-thiogalactopyranoside (IPTG). The cells were harvested by centrifugation for 15 min at 4500 rpm and 4 °C for subsequent experiments.

### Gel electrophoresis analysis

The cell pellets were re-suspended in 50 mM phosphate-buffered saline (PBS) buffer (pH 7.0). After 10 min sonication, the supernatant and precipitation were separated by centrifuging at 12,000 rpm for 10 min at 4 °C, and analyzed using sodium dodecyl sulfate–polyacrylamide gel electrophoresis (SDS-PAGE). In order to analyze the two-enzyme complex and the synthetic protein scaffold, the recombinant fusion proteins in the supernatant were purified using a fast protein liquid chromatography (GE AKTA Pure 150; General Electric Co., IA, USA) equipped with His Trap™ FF column, which was equilibrated with 50 mM imidazole buffer (50 mM phosphate buffer, 500 mM NaCl, 50 mM imidazole, pH 7.0) and eluted by using 500 mM imidazole buffer (50 mM phosphate buffer, 500 mM NaCl, 500 mM imidazole, pH 7.0). The purified proteins were concentrated, washed with 50 mM PBS buffer (pH 7.0) to remove imidazole, and verified by native polyacrylamide gel electrophoresis prepared in the absence of SDS and β-mercaptoethanol.

### Biocatalytic reaction for itaconic acid production

The biocatalytic reaction system for itaconic acid production was composed of 20 OD_600_ of the respective recombinant *E. coli* cells, 100–700 mM of citrate in 50 mM PBS buffer. To improve the itaconic acid production, Tween 80 in the range of 0–0.7% was added to the reaction system incubated at 20 °C, 25 °C, 30 °C, 35 °C, and 40 °C under the different reaction pH ranging from pH 4.0 to 8.5. Reaction samples were taken regularly for high-performance liquid chromatograph (HPLC) analysis.

### HPLC analysis

Reaction samples were analyzed using a HPLC (Agilent 1290; Agilent Technologies, Santa Clara, CA, USA), which was equipped with an HPX-87H column (300–7.8 mm, Bio-Rad, Hercules, CA, USA). HPLC was operated with an ultraviolet spectrophotometric detector to detect a 215 nm signal at 60 °C. 8 mM of sulfuric acid was used as the mobile phase at a flow rate of 0.6 mL/min.

## Supplementary Information

**Additional file 1: Table S1.** Strains and plasmids in this study. **Table S2.** Primers used in this study. **Table S3.** Mutation sites in this study. **Figure S1.** SDS-PAGE of *E. coli *BL-CAC at different IPTG concentrations and induction temperatures. **Figure S2.** Multiple sequence alignment of CAD from *A. terreus *compared with other eosinophilic strains. **Figure S3.** Relative titer of itaconic acid between recombinant *E. coli* BL-CAC and BL-CAC-DS. **Figure S4.** SDS-PAGE analysis of purified proteins (a); Lane 1 is the purified protein of recombinant strain *E. coli* BL-R-CS; Lane 2 is the purified protein of recombinant* E. coli* BL-CAC-DS; M is the Marker. Native PAGE analysis of purified proteins (b); Lane 1 is the purified protein of recombinant *E. coli *BL-R-CS, Lane 2 is the purified proteins of recombinant *E. coli *BL-CAC-DS, Lane 3 is the purified protein mixture of the recombinant *E. coli *BL-CAC-DS/R-CS incubated at 37℃ for 3 h; M is the Marker.

## Data Availability

All data generated or analyzed during this study are included in this article and its Additional file 1.
