# Supplementary material for: Construction of cell factory through combinatorial metabolic engineering for efficient production of itaconic acid
Source: Microb Cell Fact. 2022 Dec 28;21:275. doi: 10.1186/s12934-022-02001-1 (PMC9798595; doi:10.1186/s12934-022-02001-1)
Supplement: Supplementary file 1 — Additional file 1: Table S1. Strains and plasmids in this study. Table S2. Primers used in this study. Table S3. Mutation sites in this study. Figure S1. SDS-PAGE of E. coli BL-CAC at different IPTG concentrations and induction temperatures. Figure S2. Multiple sequence alignment of CAD from A. terreus compared with other eosinophilic strains. Figure S3. Relative titer of itaconic acid between recombinant E. coli BL-CAC and BL-CAC-DS. Figure S4. SDS-PAGE analysis of purified proteins (a); Lane 1 is the purified protein of recombinant strain E. coli BL-R-CS; Lane 2 is the purified protein of recombinant E. coli BL-CAC-DS; M is the Marker. Native PAGE analysis of purified proteins (b); Lane 1 is the purified protein of recombinant E. coli BL-R-CS, Lane 2 is the purified proteins of recombinant E. coli BL-CAC-DS, Lane 3 is the purified protein mixture of the recombinant E. coli BL-CAC-DS/R-CS incubated at 37℃ for 3 h; M is the Marker. [file 12934_2022_2001_MOESM1_ESM.docx]

### Additional file

**Construction of cell factory through combinatorial metabolic engineering for efficient production of itaconic acid**

Jiao Feng^1^, Chunqiu Li^1^, Hao He*^2^, Sheng Xu*^1^, Xin Wang^1^, Kequan Chen^1^

*Corresponding author: Hao He, Sheng Xu

Address: ^1^ College of Biotechnology and Pharmaceutical Engineering, Nanjing Tech University, NO.30 Puzhu Road(S), Nanjing, People’s Republic of China. PC: 211816

^2^ Petrochemical Research Insitute of Petrochina Co. Ltd., Beijing, People’s Republic of China. PC: 102206

E-mail address: hehao010@petrochina.com.cn; henryxu@njtech.edu.cn.

Table S1 Strains and plasmids in this study

| Strains | Description | References |
| --- | --- | --- |
| *E. coli* strains |  |  |
| Trans1-T1 | Used as clone strain | Transgen |
| BL21(DE3) | Used as host strain | Invitrogen |
| *Corynebacterium glutamicum* | Used as a template strain for gene cloning | Invitrogen |
| BL-CA1/AC2 | *E. coli* BL21(DE3) harboring plasmid pCDFduet-*acn1* and pACYCduet-*cadA2* | This study |
| BL-CA1/EC2 | *E. coli* BL21(DE3) harboring plasmid pCDFduet-*acn1* and pETduet-*cadA2* | This study |
| BL-CA1/RC2 | *E. coli* BL21(DE3) harboring plasmid pCDFduet-*acn1* and pRSFduet-*cadA2* | This study |
| BL-CC1/AA1 | *E. coli* BL21(DE3) harboring plasmid pCDFduet-*cadA1* and pACYCduet-*acn1* | This study |
| BL-CC1/EA1 | *E. coli* BL21(DE3) harboring plasmid pCDFduet-*cadA1* and pETduet-*acn1* | This study |
| BL-CC1/RA1 | *E. coli* BL21(DE3) harboring plasmid pCDFduet-*cadA1* and pRSFduet-*acn1* | This study |
| BL-CC2/EA1 | *E. coli* BL21(DE3) harboring plasmid pCDFduet-*cadA2* and pETduet-*acn1* | This study |
| BL-CCA | *E. coli* BL21(DE3) harboring plasmid pCDFduet-*cadA-acn* | This study |
| BL-CAC | *E. coli* BL21(DE3) harboring plasmid pCDFduet-*acn*-*cadA* | This study |
| BL-RAC | *E. coli* BL21(DE3) harboring plasmid pRSFduet-*acn*-*cadA* | This study |
| BL-CC2/AA1 | *E. coli* BL21(DE3) harboring plasmid pCDFduet-*cadA2* and pACYCduet-*acn1* | This study |
| BL-CC2/RA1 | *E. coli* BL21(DE3) harboring plasmid pCDFduet-*cadA2* and pRSFduet-*acn1* | This study |
| BL-CC | *E. coli* BL21(DE3) harboring plasmid pCDFduet- *cadA2* | This study |
| BL-C-H20E | *E. coli* BL21(DE3) harboring plasmid pCDFduet-H20E  (The 20th amino acid changed from H to E) | This study |
| BL-C-K259E | *E. coli* BL21(DE3) harboring plasmid pCDFduet-K259E  (The 259th amino acid changed from K to E) | This study |
| BL-C-H281E | *E. coli* BL21(DE3) harboring plasmid pCDFduet-H281E  (The 281th amino acid changed from H to E) | This study |
| BL-C-R323E | *E. coli* BL21(DE3) harboring plasmid pCDFduet-R323E  (The 323th amino acid changed from R to E) | This study |
| BL-C-H326E | *E. coli* BL21(DE3) harboring plasmid pCDFduet-H326E  (The 326th amino acid changed from H to E) | This study |
| BL-C-K433D | *E. coli* BL21(DE3) harboring plasmid pCDFduet-K433D  (The 433th amino acid changed from K to D) | This study |
| BL-C-R440E | *E. coli* BL21(DE3) harboring plasmid pCDFduet-R440E  (The 440th amino acid changed from R to E) | This study |
| BL-C-R470E | *E. coli* BL21(DE3) harboring plasmid pCDFduet-R470E  (The 470th amino acid changed from R to E) | This study |
| BL-CAC-DS | *E. coli* BL21(DE3) harboring plasmid pCDFduet-*acn* -Dockerin Ⅱ-*cadA*-SpyTag | This study |
| BL-R-CS | *E. coli* BL21(DE3) harboring plasmid pRSFduet- Cohesin Ⅱ- SpyCatcher | This study |
| BL-CAC-DS/R-CS | *E. coli* BL21(DE3) harboring plasmid pCDFduet-*acn* -Dockerin Ⅱ-*cadA*-SpyTag and pRSFduet- Cohesin Ⅱ- SpyCatcher | This study |
| BL-CAC-DS/R-CS2 | *E. coli* BL21(DE3) harboring plasmid pCDFduet-*acn* -Dockerin Ⅱ-*cadA*-SpyTag and pRSFduet- Cohesin Ⅱ- SpyCatche×2 | This study |
| BL-CAC-DS/R-CS3 | *E. coli* BL21(DE3) harboring plasmid pCDFduet-*acn* -Dockerin Ⅱ-*cadA*-SpyTag and pRSFduet- Cohesin Ⅱ- SpyCatche×3 | This study |
| BL-CAC-DS/A-CS | *E. coli* BL21(DE3) harboring plasmid pCDFduet-*acn* -Dockerin Ⅱ-*cadA*-SpyTag and pACYCduet- Cohesin Ⅱ- SpyCatche | This study |
| BL-CAC-DS/A-CS2 | *E. coli* BL21(DE3) harboring plasmid pCDFduet-*acn* -Dockerin Ⅱ-*cadA*-SpyTag and pACYCduet- Cohesin Ⅱ- SpyCatche×2 | This study |
| BL-CAC-DS/ A-CS3 | *E. coli* BL21(DE3) harboring plasmid pCDFduet-*acn* -Dockerin Ⅱ-*cadA*-SpyTag and pACYCduet- Cohesin Ⅱ- SpyCatche×3 | This study |
| BL-CAC-DS/E-CS | *E. coli* BL21(DE3) harboring plasmid pCDFduet-*acn* -Dockerin Ⅱ-*cadA*-SpyTag and pETduet- Cohesin Ⅱ- SpyCatche | This study |
| BL-CAC-DS/E-CS2 | *E. coli* BL21(DE3) harboring plasmid pCDFduet-*acn* -Dockerin Ⅱ-*cadA*-SpyTag and pETduet- Cohesin Ⅱ- SpyCatche×2 | This study |
| BL-CAC-DS/E-CS3 | *E. coli* BL21(DE3) harboring plasmid pCDFduet-*acn* -Dockerin Ⅱ-*cadA*-SpyTag and pETduet- Cohesin Ⅱ- SpyCatche×3 | This study |
| BL-CAR470E-DS/A-CS | *E. coli* BL21(DE3) harboring plasmid pCDFduet-*acn* -Dockerin Ⅱ-R470E-SpyTag and pACYCduet- Cohesin Ⅱ- SpyCatche | This study |
| Plasmids |  |  |
| pACYCduet-1 | expression vector, Cm^R^, P_T7_, ori | This study |
| pCDFduet-1 | expression vector, Sm^R^, P_T7_, ori | This study |
| pETduet-1 | expression vector, Amp^R^, P_T7_, ori | This study |
| pRSFduet-1 | expression vector, Km^R^, P_T7_, ori | This study |
| pETduet-Cg-*acn1* | Gene *acn* from *Corynebacterium glutamicum* insterted between *Bam*H Ⅰ and *Hind* Ⅲ sites of pETduet-1 | This study |
| pACYCduet-*acn1* | Gene *acn* from *Corynebacterium glutamicum* insterted between *Bam*H Ⅰ and *Hind* Ⅲ sites of pACYCduet-1 | This study |
| pCDFduet-*acn1* | Gene *acn* from *Corynebacterium glutamicum* insterted between *Bam*H Ⅰ and *Hind* Ⅲ sites of pCDFduet | This study |
| pRSFduet-*acn1* | Gene *acn* from *Corynebacterium glutamicum* insterted between *Bam*H Ⅰ and *Hind* Ⅲ sites of pRSFduet-1 | This study |
| pCDFduet-*cadA1* | Codon-optimized *cadA* insterted between *Bam*H Ⅰ and *Hind* Ⅲ sites of pCDFduet-1 | This study |
| pCDFduet-*cadA2* | Codon-optimized *cadA* insterted between *Nde* Ⅰ and *Kpn* Ⅰ sites of pCDFduet-1 | This study |
| pACYCduet-*cadA2* | Codon-optimized *cadA* insterted between *Nde* Ⅰ and *Kpn* Ⅰ sites of pACYCduet-1 | This study |
| pETduet-*cadA2* | Codon-optimized *cadA* insterted between *Nde* Ⅰ and *Kpn* Ⅰ sites of pETduet-1 | This study |
| pRSFduet-*cadA2* | Codon-optimized *cadA* insterted between *Nde* Ⅰ and *Kpn* Ⅰ sites of pRSFduet | This study |
| pCDFduet-*cadA-acn* | Codon-optimized *cadA* insterted between *Bam*H Ⅰ and *Hind* Ⅲ sites and gene *acn* between *Nde* Ⅰ and *Kpn* Ⅰ sites of pCDFduet-1 | This study |
| pCDFduet-*acn*-*cadA* | Gene *acn* insterted between *Bam*H Ⅰ and *Hind* Ⅲ sites and codon-optimized *cadA* insterted between *Nde* Ⅰ and *Kpn* Ⅰ sites of pCDFduet-1 | This study |
| pRSFduet-*acn*-*cadA* | Gene *acn* insterted between *Bam*H Ⅰ and *Hind* Ⅲ sites and codon-optimized *cadA* insterted between *Nde* Ⅰ and *Kpn* Ⅰ sites of pRSFduet-1 | This study |
| PCDFduet-H20E | PCDFduet-1 containing the mutant sequence of CAD that the 20th amino acid changed from H to E | This study |
| PCDFduet-K259E | PCDFduet-1 containing the mutant sequence of CAD that the 259th amino acid changed from K to E | This study |
| PCDFduet-H281E | PCDFduet-1 containing the mutant sequence of CAD that the 281th amino acid changed from H to E | This study |
| PCDFduet-R323E | PCDFduet-1 containing the mutant sequence of CAD that the 323th amino acid changed from R to E | This study |
| PCDFduet-H326E | PCDFduet-1 containing the mutant sequence of CAD that the 326th amino acid changed from H to E | This study |
| PCDFduet-K433D | PCDFduet-1 containing the mutant sequence of CAD that the 433th amino acid changed from K to D | This study |
| PCDFduet-R440E | PCDFduet-1 containing the mutant sequence of CAD that the 440th amino acid changed from R to E | This study |
| PCDFduet-R470E | PCDFduet-1 containing the mutant sequence of CAD that the 470th amino acid changed from R to E | This study |
| pCDFduet-*acn* -Dockerin Ⅱ-*cadA*-SpyTag | Genes of Dockerin Ⅱ and SpyTag inserted of pCDFduet-acn-cadA | This study |
| pRSFduet-Cohesin Ⅱ- SpyCatcher | Genes of Cohesin Ⅱ and SpyCatcher inserted into MCS1 of pRSFduet-1 | This study |
| pRSFduet-Cohesin Ⅱ- SpyCatcher×2 | Gene of Cohesin Ⅱ and two SpyCatcher segments are inserted into MCS1 of pRSFduet-1 | This study |
| pRSFduet-Cohesin Ⅱ- SpyCatcher×3 | Gene Cohesin Ⅱ and three SpyCatcher segments inserted into MCS1 of pRSFduet-1 | This study |
| pACYCduet-Cohesin Ⅱ-SpyCatcher | Genes Cohesin Ⅱ and SpyCatcher inserted into MCS1 of pACYCduet-1 | This study |
| pACYCduet-Cohesin Ⅱ-SpyCatcher×2 | Gene of Cohesin Ⅱ and two SpyCatcher segments inserted into MCS1 of pACYCduet-1 | This study |
| pACYCduet-Cohesin Ⅱ-SpyCatcher×3 | Gene of Cohesin Ⅱ and three SpyCatcher segments inserted into MCS1 of pACYCduet-1 | This study |
| pETduet-Cohesin Ⅱ- SpyCatcher | Genes of Cohesin Ⅱ and SpyCatcher inserted into MCS1 of pETduet-1 | This study |
| pETduet-Cohesin Ⅱ- SpyCatcher×2 | Genes of Cohesin Ⅱ and two SpyCatcher segments inserted into MCS1 of pETduet-1 | This study |
| pETduet-Cohesin Ⅱ- SpyCatcher×3 | Gene Cohesin Ⅱ and three SpyCatcher segments inserted into MCS1 of pETduet-1 | This study |
| pCDFduet-*acn*-Dockerin Ⅱ-R470E-SpyTag | R470E fragment replaced the CAD fragment | This study |

Table S2 Primers used in this study

| Name | Primer sequence (5′–3′) |
| --- | --- |
| Cg-*Bam*H I-F | CGGGATCCGAGCTCACTGTGACTGAAAGCAAGA |
| Cg-*Hind* III-R | CCCAAGCTTCTTAGAAGAAGCAGCCATCTGACG |
| *cadA*-*Bam*H I-F | CGGGATCCATGACCAAACAGAGCGCCGAT |
| *cadA*-*Hind* III*-*R | CCCAAGCTTAACCAGTGGAGATTTAACCGGACA |
| *cadA*-*Nde* I-F | GGGTTTCATATGATGACCAAACAGAGCGCCGAT |
| *cadA*-*Kpn* I*-*R | GGGGTACCAACCAGTGGAGATTTAACCGGACA |
| H20E-F | ACCAGCGAAATTTGT**GAA**TGGGCCAGCAAC |
| H20E-R | TTTGCCCTGTTCTGCCAGAACTTC |
| K259E-F | CTGAAAATGTTTACC**GAA**GGTAATGGTCGTGAACCT |
| K259E-R | ATCAAATTCGCTAAACTGACTCAGCAGACACTGCTG |
| H281E-F | CTGGGTAGCTTTTGG**GAA**ACCTTTACCATTCGT |
| H281E-R | GCTACTGGTAACTTTACGTGCCAGATC |
| R323E-F | GCGAATCTGAGCAATATT**GAA**CATGTTCATGTGCAGCTG |
| R323E-R | CGGCATTGGTTCTTTAACACCCAG |
| H326E-F | AGCAATATTCGTCATGTT**GAA**GTGCAGCTGAGTACC |
| H326E-R | GCAGAATACGTTCATTCGGCATTGGTTCTTTAAC |
| K433D-F | AAACCGCTGGGTGTT**GAT**GAACCAATGCCGAAT |
| K433D-R | CAAAAAACCCCTCAAGACCCGTTT |
| R440E-F | GAACCAATGCCGAATGAA**GAA**ATTCTGCATAAATATCGTACCCTG |
| R440E-R | CGTGTGCTTCTCAAATGCCTGAGG |
| R470E-F | CTGGTGCTGGGCCTGGAT**GAA**CTGACAGATATTAGC |
| R470E*-*R | GGTTCAGGGCAGGGTCGTTAAAT |
| D2-Linker1-F | GGCGGAGGTGGCTCTGGCGGTGGCGGATCGGGATCCCTGCAGAATAAACCGG |
| D2-Linker1-R | CTGTGCATCATAATCGCTACTGGTTG |
| D2-tong-F | AGATGGCTGCTTCTTCTAAGGGCGGAGGTGGCTCT |
| D2-tong-R | TTATGCGGCCGCAAGCTTTACTGTGCATCATAATCGCTACTGGT |
| CDF-acn-F | TAAAGCTTGCGGCCGCATAATG |
| CDF-acn-R | CTTAGAAGAAGCAGCCATCTGACG |
| St-Linker2-F | GGTTCAGGGGGATCCGGTGTCGACGGCTCAGGTGCACATATCGTCATG |
| St-Linker2-R | TCCGCTGCCTTTGGTCGGTTT |
| St-tong-F | CGGTTAAATCTCCACTGGTTGGTTCAGGGGGATCCGGT |
| St-tong-R | TTTACCAGACTCGAGGGTTATCCGCTGCCTTTGGTCG |
| CDF-cadA-F | TAACCCTCGAGTCTGGTAAAGAAACC |
| CDF-cadA-R | AACCAGTGGAGATTTAACCGGAC |
| 6H-cadA-tong-F | AAGAAGGAGATATACATATGCATCACCATCATCACCACACCAAAC |
| 6H-cadA-F | CATCACCATCATCACCACACCAAACAGAGCGCCGATAGC |
| RSF-F | GAGCTCGGCGCGCCTGCAGGT |
| RSF-R | TGAATTCGGATCCTGGCTGTGGT |
| RSF-C2-F | CACAGCCAGGATCCGAATTCAATGGAAGCAACCCCGAGTATTGA |
| RSF-C2-R | GGCCGCATCTTTCAGCGG |
| RSF-Sc-Linker1-F | GGCGGAGGTGGCTCTGGCGGTGGCGGATCGGGCGGGAGCGCTATGGTG |
| RSF-Sc-Linker1-R | GTCAATATGGGCATCCCCTTTCGT |
| RSF-Sc-tong-F | AACCGCTGAAAGATGCGGCCGGCGGAGGTGGCTCTG |
| RSF-Sc-tong-R | ACCTGCAGGCGCGCCGAGCTCGTCAATATGGGCATCCCCTTT |
| RSF-F2 | GAGCTCGGCGCGCCTGCAGGT |
| RSF-R2 | GGCCGCATCTTTCAGCGG |
| RSF-Sc×2-Linker2-F | GGTTCAGGGGGATCCGGTGTCGACGGCGGGAGCGCTATGGTGGAC |
| RSF-Sc×2-Linker2-R | GTCAATATGGGCATCCCCTTTCGTAGCTTTCCC |
| RSF-Sc×2-tong-F | AAGGGGATGCCCATATTGACGGTTCAGGGGGATCCGGT |
| RSF-Sc×2-tong-R | ACCTGCAGGCGCGCCGAGCTCGTCAATATGGGCATCCCCTTT |
| RSF-F3 | GAGCTCGGCGCGCCTGCAGGT |
| RSF-R3 | GTCAATATGGGCATCCCCTTTCGTAGCTTTCCC |
| RSF-Sc×3-Linker3-F | GGTTCTGGTTCTGGTTCTGGTTCTGGTTCTGGTTCTGGCGGGAGCGCTATGGTGGAC |
| RSF-Sc×3-Linker3-R | GTCAATATGGGCATCCCCTTTCGTAGCTTTCCC |
| RSF-Sc×3-tong-F | AACCGCTGAAAGATGCGGCCGGTTCTGGTTCTGGTTCTGGTTCTGGT |
| RSF-Sc×3-tong-R | CCGCCAGAGCCACCTCCGCCGTCAATATGGGCATCCCCTTTCG |
| RSF-F4 | GGCGGAGGTGGCTCTGG |
| RSF-R4 | GGCCGCATCTTTCAGCGG |
| *cadA*-R470E-F | CTGGTGCTGGGCCTGGATGAACTGACAGATATTAGC |
| *cadA*-R470E-R | CAGGCCCAGCACCAGATCTTC |

Table S3 Mutation sites in this study

| Amino acid of CAD | Conservation (%) | Acidic amino acid | Mutation |
| --- | --- | --- | --- |
| H20 | 35 | D, E×4, Q×2 | H20E |
| K259 | 21 | E×8 D×3 | K259E |
| H281 | 36 | E×6 | H281E |
| R323 | 51 | D, E×5 | R323E |
| H326 | 31 | D, E×5 | H326E |
| K433 | 32 | D×5, E | K433D |
| R440 | 46 | Q, E×7 | R440E |
| R470 | 33 | Q, E×5 | R470E |


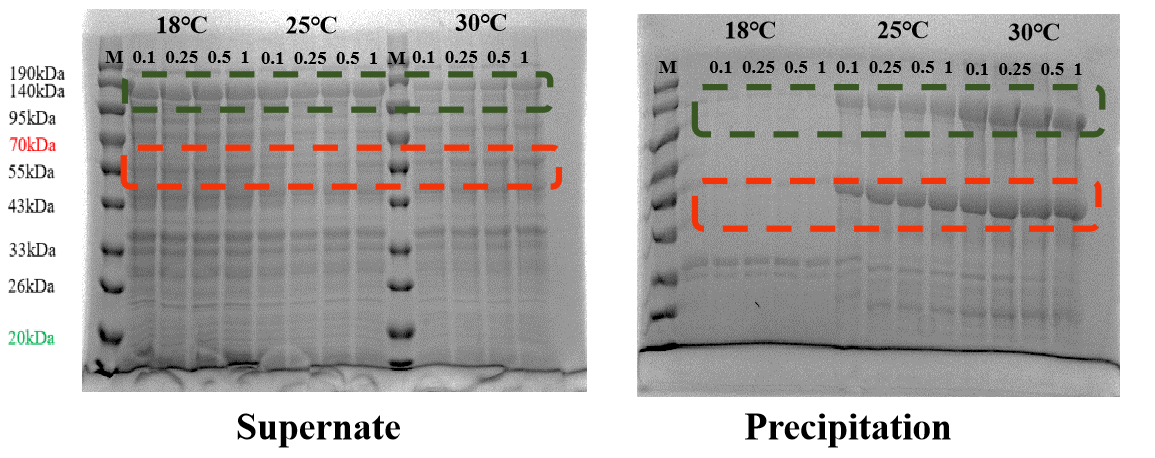


Figure S1 SDS-PAGE of *E. coli* BL-CAC at different IPTG concentrations and induction temperatures


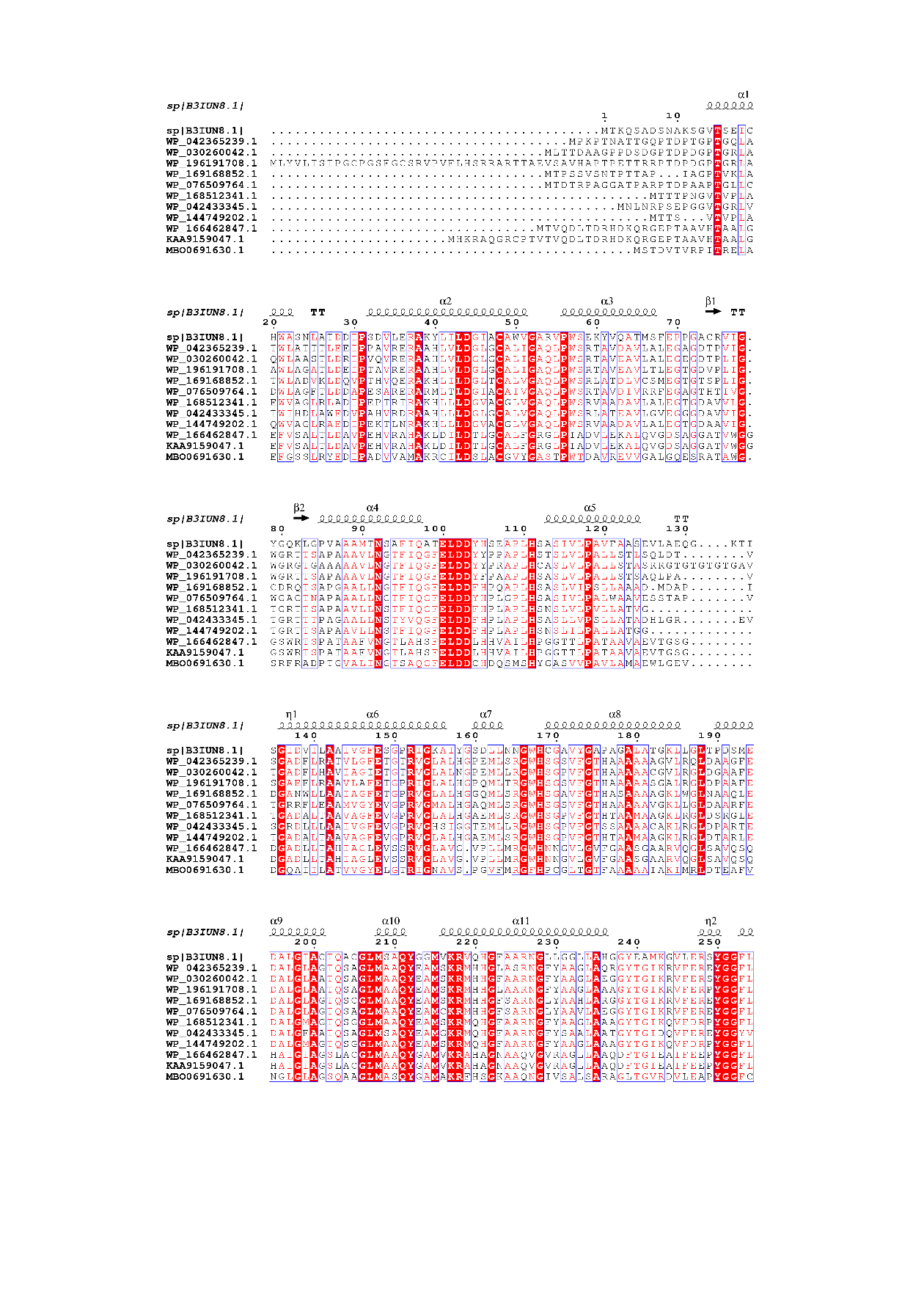


Figure S2 Multiple sequence alignment of CAD from *A. terreus* compared with other eosinophilic strains





Figure S3 Relative titer of itaconic acid between recombinant *E. coli* BL-CAC and BL-CAC-DS


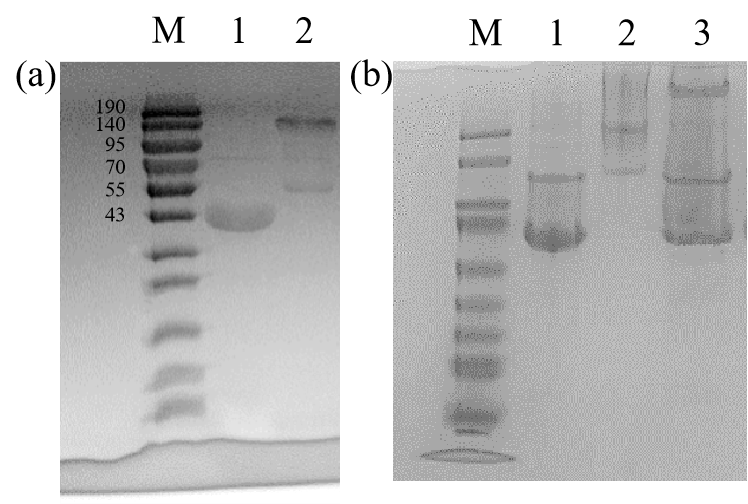


Figure S4 SDS-PAGE analysis of purified proteins (a); Lane 1 is the purified protein of recombinant strain *E. coli* BL-R-CS; Lane 2 is the purified protein of recombinant *E. coli* BL-CAC-DS; M is the Marker. Native PAGE analysis of purified proteins (b); Lane 1 is the purified protein of recombinant *E. coli* BL-R-CS, Lane 2 is the purified proteins of recombinant *E. coli* BL-CAC-DS, Lane 3 is the purified protein mixture of the recombinant *E. coli* BL-CAC-DS/R-CS incubated at 37℃ for 3 h; M is the Marker.
